# Seasonal drought shapes the relationship between stem CO_2_ efflux and belowground respiration in an even-age rubber plantation on Hainan Island, China

**DOI:** 10.3389/fpls.2025.1552859

**Published:** 2025-07-16

**Authors:** Xinwei Guo, Bo Song, Rui Sun, Guoyu Lan, Bangqian Chen, Chuan Yang, Zhixiang Wu

**Affiliations:** ^1^ Rubber Research Institute, Chinese Academy of Tropical Agricultural Sciences, Haikou, China; ^2^ School of Accounting, Hainan Vocational University of Science and Technology, Haikou, Hainan, China; ^3^ Hainan Danzhou Tropical Agro-ecosystem National Observation and Research Station, Ministry of Science and Technology of the People's Republic of China, Danzhou, China

**Keywords:** carbon balance, xylem sap, tapping activities, leaf area index, growth phenology

## Abstract

**Introduction:**

Stem CO_2_ efflux (*E_c_*) reflects the amount of photo-assimilated carbon released back into the atmosphere and plays a critical role in the carbon balance of tree and forest ecosystems. Despite previous studies indicating that a portion of stem CO_2_ originates from root respiration (*R_root_*), the seasonal dynamics of *E_c_* and its relationship with belowground respiration remain poorly understood, particularly in tropical regions characterized by distinct dry and wet seasons.

**Methods:**

To address this gap, we investigated *E_c_* in tapping and non-tapped rubber trees, along with environmental factors and physiological traits (sap flow flux density, root respiration, and leaf area index) from 2018 to 2021.

**Results:**

Our results showed that tapping activity increased the *E_c_* of rubber trees compared to non-tapped trees, with increases ranging from 10.37% to 233.66%. However, the magnitude of this increase varied between the dry and wet seasons. Although tapping enhanced the *E_c_*, it did not alter the *E_c_* seasonal pattern. Consequently, *E_c_* in both tapped and non-tapped rubber trees displayed an overall single-peak pattern, with significantly lower values during the dry season compared to the wet season, suggesting growth phenology primarily regulates *E_c_* seasonal dynamics. Structural equation modeling revealed that root respiration (*R_root_*), sap flow flux density (*F_d_*), and soil moisture at 50 cm depth as the primary drivers of the *E_c_* variations during the dry season. In contrast, soil moisture at 5 cm depth and air temperature (*T_a_*) were identified as dominant factors influencing *E_c_* in the wet season, with belowground respiration having a negligible influence.

**Discussion:**

These results suggest that the relationship between *E_c_* and belowground respiration is environmentally sensitive and exhibits seasonal dependency.

## 1 Introduction

Autotrophic respiration is a principal process regulating how tree growth and ecosystem productivity respond to environmental changes, such as variations in atmospheric CO_2_ levels, air temperature and moisture (Ryan et al., 1997; Robertson et al., 2010; Tarvainen et al., 2014; Zhao et al., 2018a). Tree stems, as a substantial source of autotrophic respiration, can release a comparable amount of CO_2_ to emissions from leaves (Trumbore et al., 2013; Zhao et al., 2018b). Stem CO_2_ efflux (*E*_c_) from respiratory processes is related to different tree function maintenance and comprises 5-42% of the total ecosystem respiration (Rodríguez-Calcerrada et al., 2015; O’Leary et al., 2019; Helm et al., 2023). Understanding *E*_c_ is crucial for predicting ecosystem carbon budgets, particularly in the context of climate change. However, there is a lack of information regarding how environmental and physiological factors regulate the variability in *E*_c_.

Variability of *E*_c_ depends on a complex set of factors, including environmental conditions (such as temperature, soil pH, water availability) and tree physiological traits such as leaf area index (LAI), sap velocity, root respiration, nonstructural carbohydrates content, and stem photosynthetic activity (Teskey et al., 2008; Wertin and Teskey, 2008; Cerasoli et al., 2009; Bužková et al., 2015; Rodríguez-Calcerrada et al., 2015; Aubrey and Teskey, 2021; Noh et al., 2021; Song et al., 2023). Water availability and temperature are widely recognized as key environmental drivers of stem CO_2_ efflux. Water availability impacts cell turgor pressure, growth, and phloem and sapwood transport, while temperature affects respiratory enzymes, CO_2_ solubility in xylem sap, and radial CO_2_ diffusion rates (Han et al., 2017; Noh et al., 2021). Generally, *E*_c_ typically decreases with declining soil moisture and temperature. However, in other cases, the relationships between *E*_c_ and soil water content and temperature were less clear or could not be definitely established (Saveyn et al., 2008a; Guidolotti et al., 2013; Rodríguez-Calcerrada et al., 2015; Jones et al., 2024). The observed discrepancies may be accounted for by differences in tree physiological state. Sap flow, stem photosynthesis, and nonstructural carbohydrates (NSC) availability could potentially impact *E*_c_ (Bloemen et al., 2013a; Trumbore et al., 2013; Jones et al., 2024). Given the high CO_2_ solubility in water, a portion of the produced through stem respired CO_2_ may dissolve in the xylem sap and be transported via transpiration stream rather than diffusing radially (Saveyn et al., 2008b; Teskey et al., 2008; Edwards and Wullschleger, 2021; Helm et al., 2023). In tree stems, internal CO_2_ originating from respiration can also be assimilated by active chloroplasts located within the inner bark and xylem. Stem photosynthesis has the capacity to absorb 7%-123% of the CO_2_ that is respired (Avila et al., 2014). NSC reserves serve as a substrate for respiration and their content levels in the stem are closely linked to the CO_2_ efflux. Since NSC responses to environmental shifts are complex, they may explain discrepancies in relationships between *E*_c_ and environmental factors (Maier et al., 2010; Jones et al., 2024). Additionally, another potential source of *E_c_* is CO_2_ released by root respiration and transported upwards by the transpiration stream (Teskey et al., 2008; Aubrey and Teskey, 2021). Although the stem CO_2_ efflux may be influenced by the CO_2_ transport in the transpiration stream. However, the contribution of root respiration to *E_c_* has always been overlooked in previous eco-physiological studies.

Rubber plantations are the predominant type of man-made forests in tropical regions in China (Song et al., 2023). In contrast to the Amazon rainforest zone, the birthplace of rubber trees, rubber cultivation areas in China experience a tropical monsoon climate characterized by distinct wet seasons (high temperature and abundant precipitation) and dry seasons (low temperature and reduced precipitation). These seasonal variations significantly impact the trees physiological state (Chen and Cao, 2015; Lai et al., 2023), potentially altering stem respiration. Song et al. (2023) confirmed clear seasonal dynamics in the stem CO_2_ efflux of rubber trees. Yet, the impacts of sap flow and root respiration on stem CO_2_ efflux under fluctuating environments remain understudied, restricting a mechanistic understanding of its variability. Additionally, rubber trees, as the primary source of natural rubber, latex harvest require a tapping process involving bark incision and laticifer disruption (Zhang et al., 2016). This specific activity may directly or indirectly alter tree physiology, thereby affecting *E*_c_. Sucrose serves as a precursor for latex biosynthesis; tapping can shift photosynthetic carbon allocation, with a considerable portion of the assimilated carbon being stored as non-structural carbohydrates at the tapped panel of the rubber trees to support latex regeneration (Duangngam et al., 2020). Such NSC accumulation supplies substrates for stem respiration (Maier et al., 2010). Concurrently, tapping activities also reduce the sap flow rate in the stem (Kunjet et al., 2013), thereby influencing the transport of root-respired CO_2_ via the transpiration stream and its contribution to *E*_c_ (Trumbore et al., 2013; Dukat et al., 2024). Furthermore, compared to virgin bark, regenerated bark post-tapping contains a higher proportion of soft tissue, which enhances radial CO_2_ diffusion (Gopal and Thomas, 2014). Theoretically, these physiological changes should elevate *E*_c_. However, empirical studies report no significant difference in *E_c_* between the tapped and non-tapped panels of rubber trees (Yan et al., 2009), suggesting that environmental constraints (e.g., seasonal drought) may mask tapping effects. Thus, further investigation into tapping’s impact on *E*_c_ across seasonal gradients is warranted.

In this experiment, we sought to explore the seasonal dynamics of stem CO_2_ efflux and the underlying biophysical mechanism. We hypothesized that (1) tapping activities would enhance stem CO_2_ efflux due to altered carbon allocation and bark regeneration, with the magnitude of increase varying significantly between dry and wet seasons; (2) A significant positive correlation would exist between root respiration and stem CO_2_ efflux during dry seasons, attributed to reduced transpiration rates prolonging retention of CO_2_ derived from root respiration in xylem sap, thereby enhancing its radial diffusion from stems (Dukat et al., 2024).

## 2 Materials and methods

### 2.1 Site description

**Figure 1:**
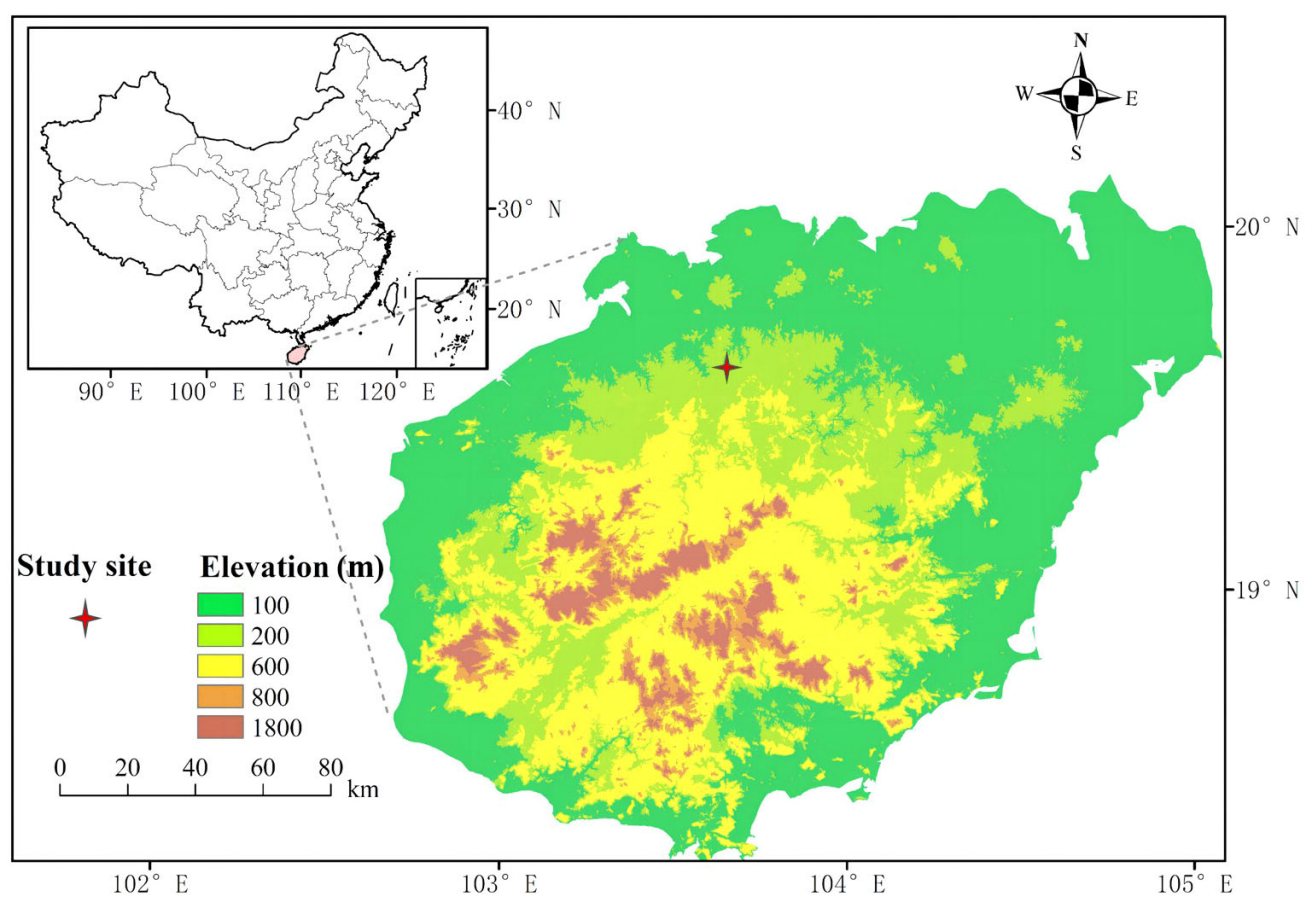
Location of the study site.

The experiment was conducted in a managed pure even-aged rubber plantation at Danzhou Tropical Agro-ecosystem National Observation and Research Station (19^°^31’N, 109^°^28’E; altitude 114 m a.s.l.) in Danzhou, Hainan south China (**Figure 1**). Mean annual precipitation, relative air humidity and temperature are ca. 1607 mm, ca. 83% and ca. 24.1 °C, respectively (Yang et al., 2023). The experiment site experiences a typical tropical monsoon climate with strong seasonal rainfall variability featuring distinct wet seasons (May to Sep) and drought seasons (Oct to Apr). The soil type is classified as latosol, with an average soil depth of approximately 1 m, predominantly sandy clay loam texture.

The rubber trees (*Hevea brasiliensis* (Willd. ex A. Juss.) Muell. Arg., clone 7-33-97) were planted in 2001 at a density of 476 trees ha^-1^. In 2021, sampled trees had a mean height of ca. 17.53 m and a stem diameter (DBH) of ca. 0.23 m at 1.5 m. The selected sample trees were divided into two treatment groups: tapping rubber trees and non-tapped rubber trees, with a subset instrumented for sap flow monitoring (**Table 1**). Tapping occurred from May to December following regional practice. Tapping cut length is standardized at half of the trunk circumference (S/2 system), with a downward-oriented cutting direction at an inclination angle of 25-30°. Cutting depth is precisely controlled at 1.2-1.8 mm above the cambium layer, and the tapping frequency is maintained at once every three days.

**Table 1:** Characteristics of the sampled trees for stem CO_2_ efflux and sap flow measurements.

Treatment	Tree	Height(m)	Diameter of stem at 1.5 m (cm)
Tapping	TJ301	16.9	20.5
Tapping	TJ302	16.6	19.7
Tapping	TJ303	18.5	22.3
Non-tapping	UT201	17.1	22.6
Non-tapping	UT202	18.2	24.8
Non-tapping	UT203	17.8	21.6
Sap flow	I101	17.6	22.6
Sap flow	I102	18.4	24.8
Sap flow	I103	16.7	23.6

TJ, UT and I represent tree numbers.

### 2.2 Environmental factors and leaf area index measurements

Environmental data were collected from a 50 m flux tower at the study site. The tower was equipped with multiple sensors to monitor environmental factors, such as air temperature, relative humidity, soil temperature, and moisture. More detailed information, including equipment model, installation height, and data recording frequency, can be found in the reference (Yang et al., 2023). Canopy leaf area index (LAI) was measured twice a month using a canopy analyzer (LAI-2200C, LI-Cor Inc., Lincoln, NE, USA).

### 2.3 Sap flux density measurements

Sap flux density (*F_d_*) was measured in three non-tapped rubber trees (**Table 1**) with the thermal dissipation method (Granier, 1987). Each sensor was comprised of a pair of probes (20 mm long and 2 mm in diameter), with one probe heated at a constant power of 0.2 W and the other unheated serving as a temperature reference. These probes were inserted into the sapwood at 20–30 mm depth, with 10 cm vertical separation at 1.5 m height. Voltage difference between probes was converted into temperature, recorded at half an hour by CR10X data logger (Campbell Scientific Inc., USA). Rubber trees are diffuse-porous species, thus, it is appropriate to use Granier’s empirical equation (Granier, 1987):


$$F_{d}=119*{\left(\frac{\Delta {\text{T}}_{m}-\Delta \text{T}}{\Delta \text{T}}\right)}^{1.231}$$

Where *F_d_* is a sap flux density (g·m^-2^s^-1^), △T*_m_* is the maximum temperature difference between day and night (°C), △T is the instantaneous temperature difference (°C).

### 2.4 Root respiration measurements

**Figure 2:**
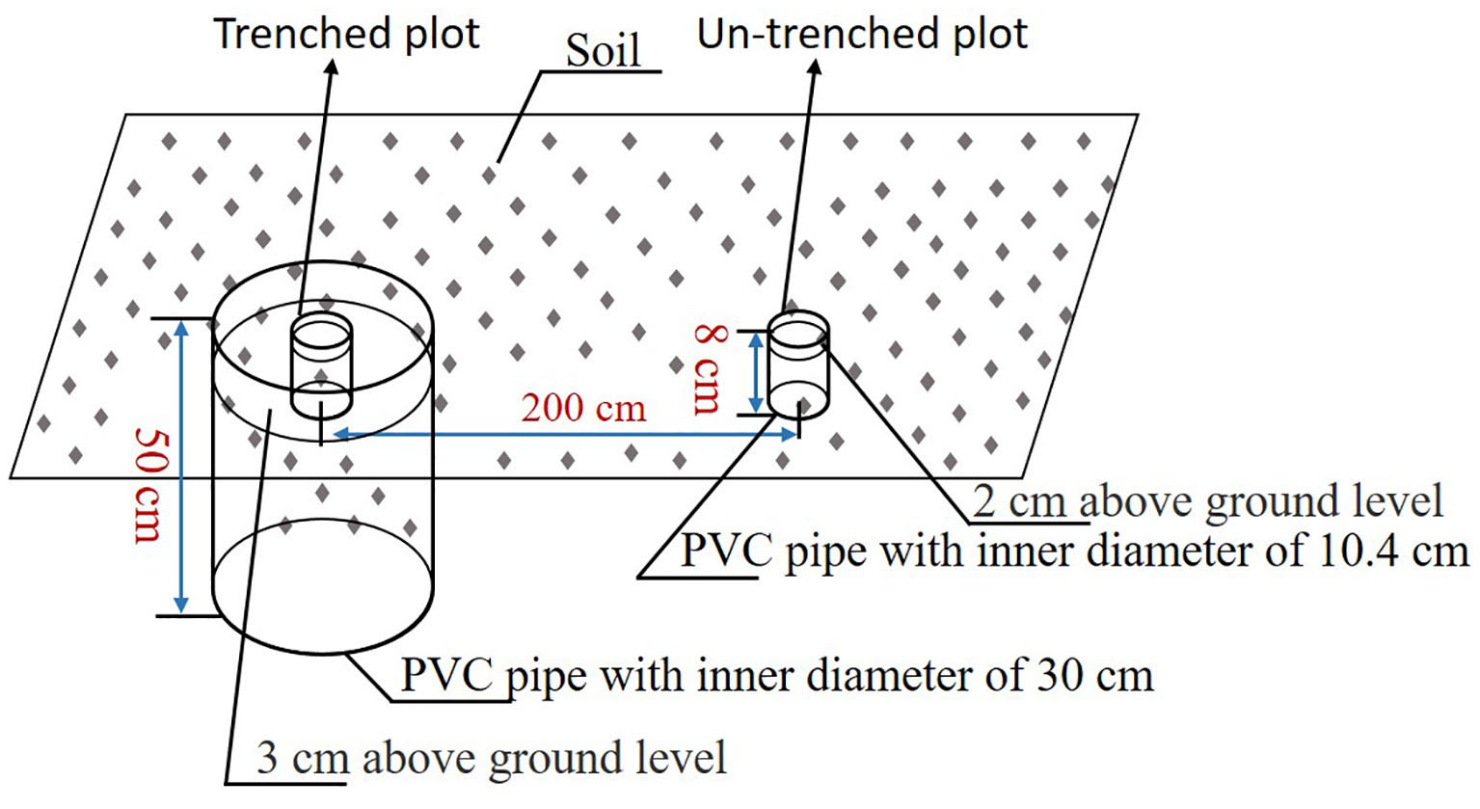
Experimental design and measurement of belowground respiration.


Root respiration (*R_root_*) was estimated using a modified trenching method. In May 2018, five paired plots (trenched vs. non-trenched) were established. Based on root distribution (predominantly 0–40 cm), the trenched plots were excavated to 50 cm depth for root exclusion. The total soil respiration (*S_r_*) and heterotrophic respiration (*H_r_*) were measured in the non-trenched and trenched plots, respectively. At the non-trenched plots, PVC soil collars (10.2 cm inner diameter × 8 cm height) were vertically inserted to a depth of 6 cm into the soil, leaving approximately 2 cm protruding aboveground. For the trenched plots, two concentric collars were deployed, an inner collar matching the non-trenched specifications (10.4 cm diameter × 8 cm height), and an outer barrier collar (30 cm inner diameter × 50 cm height) inserted to a depth of 47 cm. This nested design achieved dual objectives to completely exclude lateral root ingrowth via the outer collar’s subsoil isolation, and to minimize soil structural disturbance within the inner sampling zone. Paired plots were spatially separated by 200 cm to avoid cross-contamination. Collars were installed 6 months pre-measurements to allow root decomposition and soil stabilization (**Figure 2**). From 2019-2021, *S_r_* and *H_r_* were measured twice once a month using a portable infra-red gas analyzer equipped with a 09-soil flux chamber (LI-6400, Li-Cor Inc.). Soil temperature at 0–5 cm was measured simultaneously using the 09-soil flux chamber with a soil temperature probe. *R_root_* was calculated as the difference between *S_r_* and *H_r_* as follows:

$$R_{root}=S_{r}-H_{r}$$

### 2.5 Experimental design and stem CO_2_ efflux measurement

**Figure 3:**
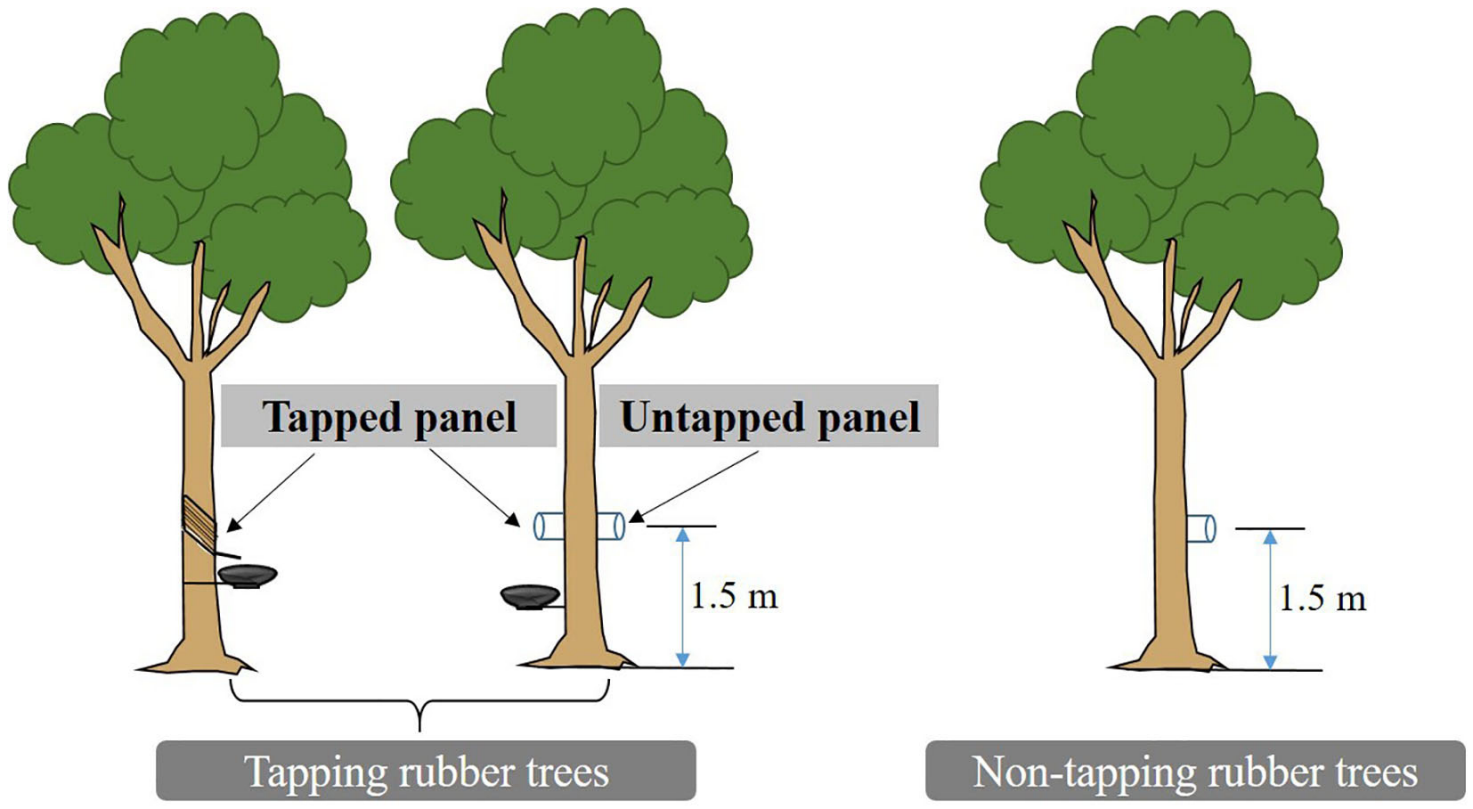
Experimental design and measurement of stem CO_2_ efflux in rubber trees.

Stem CO_2_ efflux (*E*_c_) at 9:00-11:00 am was measured *in situ* twice a month from 2018 to 2021 using a portable infrared gas analyzer (LI-6400, Li-Cor Inc.) (Darenova et al., 2024; Mills et al., 2024). Three tapped and non-tapped rubber trees with compared DBH and height were selected. Two home-made PVC collars (10.4 cm inner diameter, 8 cm height) were installed on the tapped panel and untapped panel of tapped trees at 1.5 m height above the ground. The height and direction of the PVC collar installation on the non-tapped rubber tree matched that of the untapped panel of the tapping rubber tree (**Figure 3**). To avoid the influence of atmosphere air on measurement results, glue (100% neutral transparent waterproof silicone) was used in the connection location between the stem and PVC collar.

*E*_c_ was measured per unit surface area of the stem. Since the stem surface was not flat, the collar-covered area and air enclosed in the chamber differed among plots. Therefore, it is necessary to calibrate the obtained value with the following equation (Xu et al., 2011):


$$E_{c}^{\prime }=\frac{V+V\prime -\pi {\left(\frac{d}{2}\right)}^{2}h}{V}*\frac{S}{S\prime }*E_{c}$$

*E*_c_ and *E*_c_’, are the stem area-based CO_2_ release rates (μmol·m^-2^s^-1^) before and after calibration, respectively; V and V’, the default values of the systemic chamber and actual value of the collar volume (cm^3^); S and S’, the default values of the systemic chamber and actual value of surface area the collar (cm^2^). D and h; the external diameter and the depth of the collar chamber (cm), respectively.

### 2.6 Data processing and analysis

In this study, we initially utilized data collected from 2018 to 2021 to explore the seasonal patterns of *E_c_*. Restricted Maximum Likelihood (REML) linear mixed-effects models (LMMs) were fitted using the lme function in R (nlme package; Pinheiro et al., 2013) to assess the effects of treatments, sampling times, and their interaction on *E_c_*, with treatment and time as fixed effects and tree identity as a random intercept. Subsequent analyses focused exclusively on 2019–2020, as data from both dry and wet seasons were available during this interval. Following Yang et al. (2023), data were grouped by season, and LMMs were tested for treatment and seasonal differences in *E_c_*. Linear regression analyzed relationships between *E_c_* and environmental/physiological factors during each season. To ensure comparability, all factors were measured daily between 09:00–11:00 (aligned with *E_c_* measurements). Since tapping reduces sap flow flux density (*F_d_*) but not its diurnal/seasonal trends in rubber forests (Kunjet et al., 2013), we used *F_d_* from non-tapped trees to assess correlations with *E_c_*. This approach minimizes confounding effects on correlation analyses. Finally, a piecewise structural equation model (SEM; piecewiseSEM R package; Lefcheck, 2016) was built using control (CK; non-tapped) trees to evaluate interactions among variables. This data analysis is to ensured completeness of physiological data (e.g., *F_d_*), and avoidance of tapping-induced confounding of *E_c_*. Multicollinearity was controlled by calculating variance inflation factors (VIF; vif function, car package). Predictors with VIF > 5 were excluded (Santibáñez-Andrade et al., 2015). Remaining variables were selected based on biological relevance, balancing model parsimony and explanatory power. Model fit was assessed via Fisher’s C statistic (*p* > 0.05) and AIC.

## 3 Results

### 3.1 Seasonal dynamics in environmental and physiological variables

**Figure 4:**
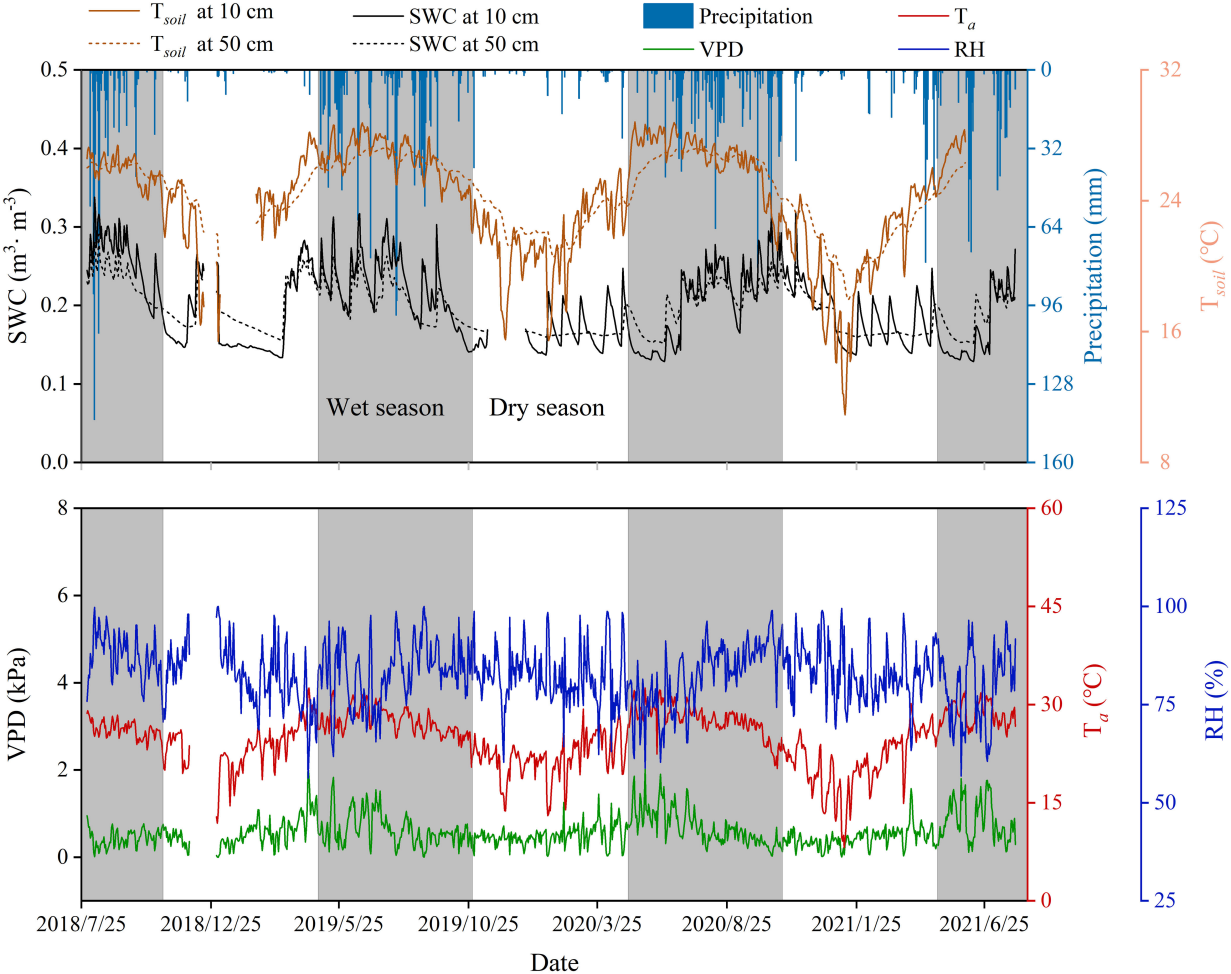
Variation of daily mean environmental variables. SWC, soil moisture; RH, relative humidity; VPD, vapor pressure deficit; *T_a_*, air temperature; *T_soil_*, soil temperature. The gray area and blank area in the figure represent the wet season and dry season respectively, the same below.

Precipitation distribution was uneven throughout the year, with 85.6-92.7% of annual totals occurring from May to October. Soil temperature (*T_soil_*), moisture (SWC), air temperature (*T_a_*), relative humidity (RH), and vapor pressure deficit (VPD) exhibited unimodal seasonal patterns. The study area exhibited a concurrent hot-wet season climate, with significantly higher temperature and soil moisture during the wet season than the dry season (*p* < 0.05; **Figure 4**).

**Figure 5:**
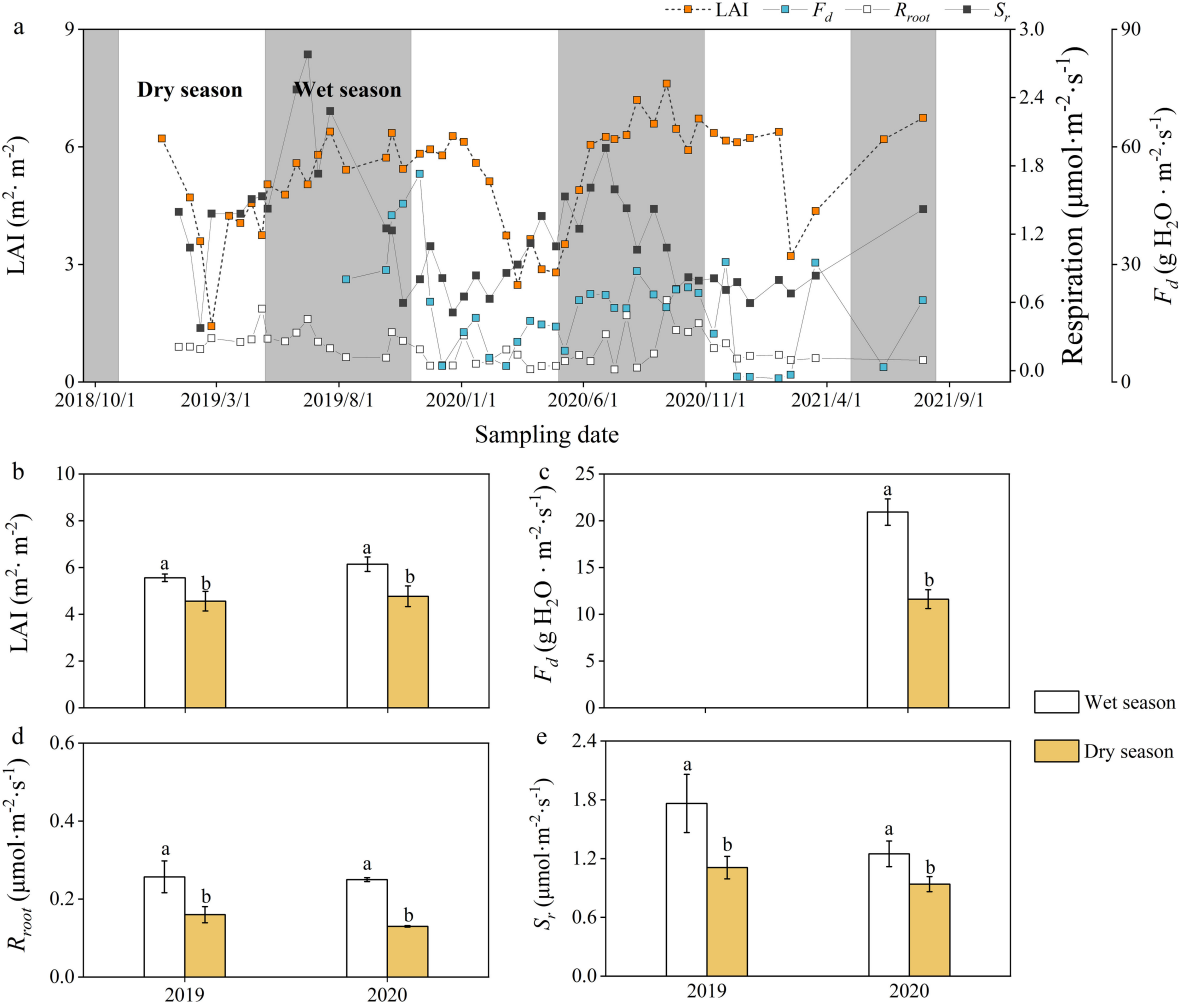
Daily **(a)** and seasonal **(b–e)** variations in physiological factors and soil respiration. LAI, leaf area index; *F_d_*, sap flux density; *R_root_*, root respiratory; *S_r_*, soil respiration. Values are means ± SE. Lowercase letters indicate a significant difference between the wet season and the dry season (*p* < 0.05).

In terms of physiological variables, the leaf area index (LAI), sap flow flux density (*F_d_*), and root respiration (*R_root_*) also presented single-peak seasonal variation, with interannual variation in peak timing (**Figure 5a**). Compared with dry seasons, rubber trees have higher LAI, *F_d_* and *R_root_* in the wet season (*p* < 0.05; **Figures 5b-d**). Soil respiration (*S_r_*) showed distinct seasonality, with wet season rates significantly exceeding dry season values (p < 0.05; **Figure 5e**).

### 3.2 Seasonal dynamics in stem CO_2_ efflux of the rubber trees

**Figure 6:**
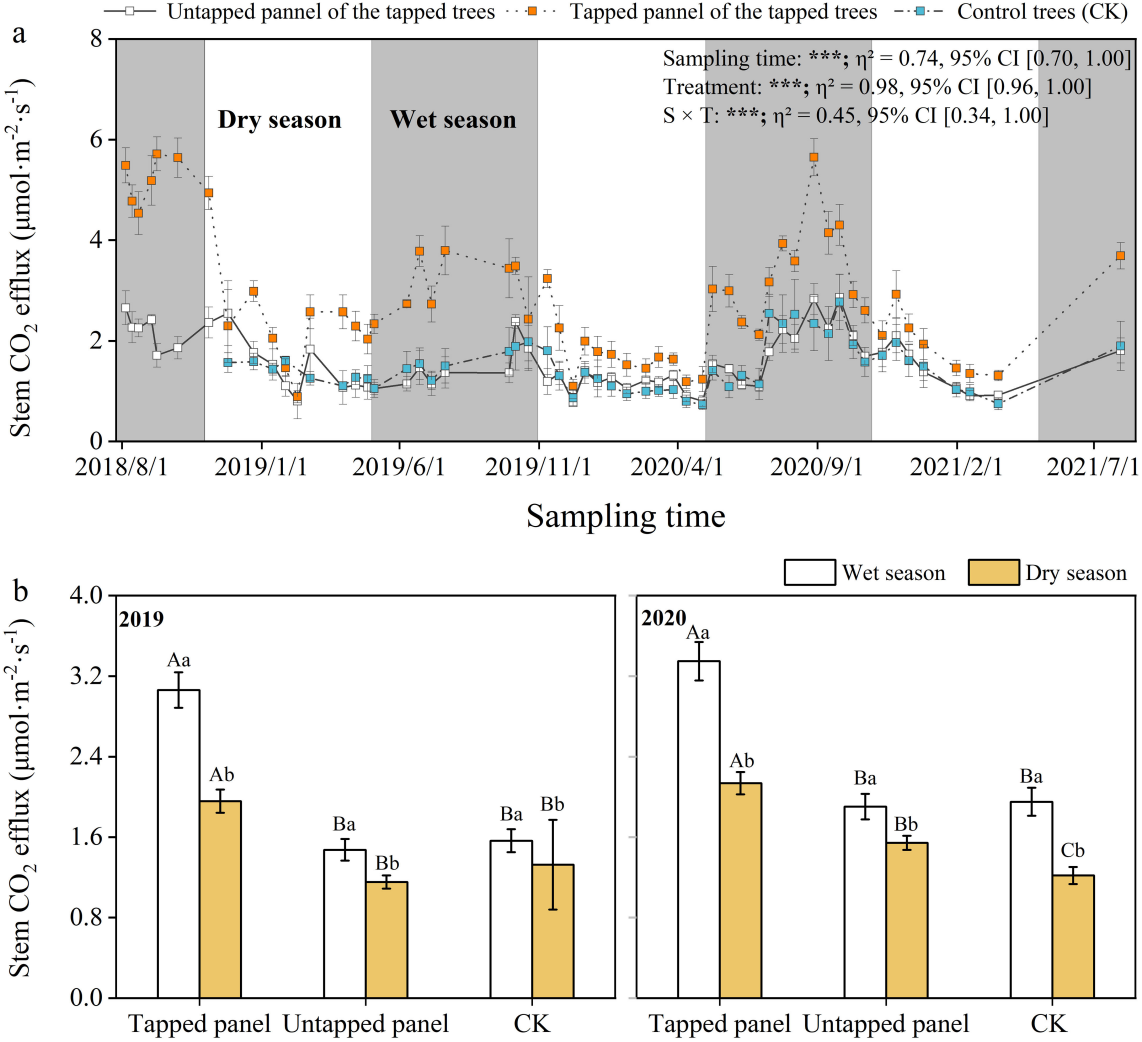
Daily and seasonal variation in stem CO_2_ efflux (*E_c_*). Values are means ± SE. Lowercase letters indicate a significant difference between the wet season and the dry season, Capital letters indicate differences between treatments (*p* < 0.05). The effects of treatment, sampling time, and their interaction on *E_c_* are displayed in the figure. (n.s., *p* > 0.05; ***p < 0.001; **p < 0.01; *p < 0.05).

Seasonal patterns in *E*_c_ were consistent across tapping treatments, mirroring temperature and moisture trends (**Figure 6a**). Rubber tree *E*_c_ in the wet seasons was significantly higher than the dry seasons (*p* < 0.05; **Figures 6b, c**). *E*_c_ at the tapped panel of tapping rubber trees ranged between 0.89 and 5.71 μmol·m^-2^·s^-1^, while the untapped panel of tapping and non-tapped rubber trees ranged from 0.77 to 2.86, 0.73 to 2.77 μmol·m^-2^·s^-1^, respectively. Compared with non-tapped treatment (CK), tapping significantly increased *E*_c_ at the tapped panel (10.37%-233.66%), but did not affect the untapped panel. The minimum difference between *E*_c_ on the tapped panel and the untapped panel occurred during dry seasons (e.g., February), while maxima aligned with the wet seasons, though peak months varied interannually (**Figure 6a**).

### 3.3 Variation in the relationships between *E_c_* and environmental and physiological factors

**Figure 7:**
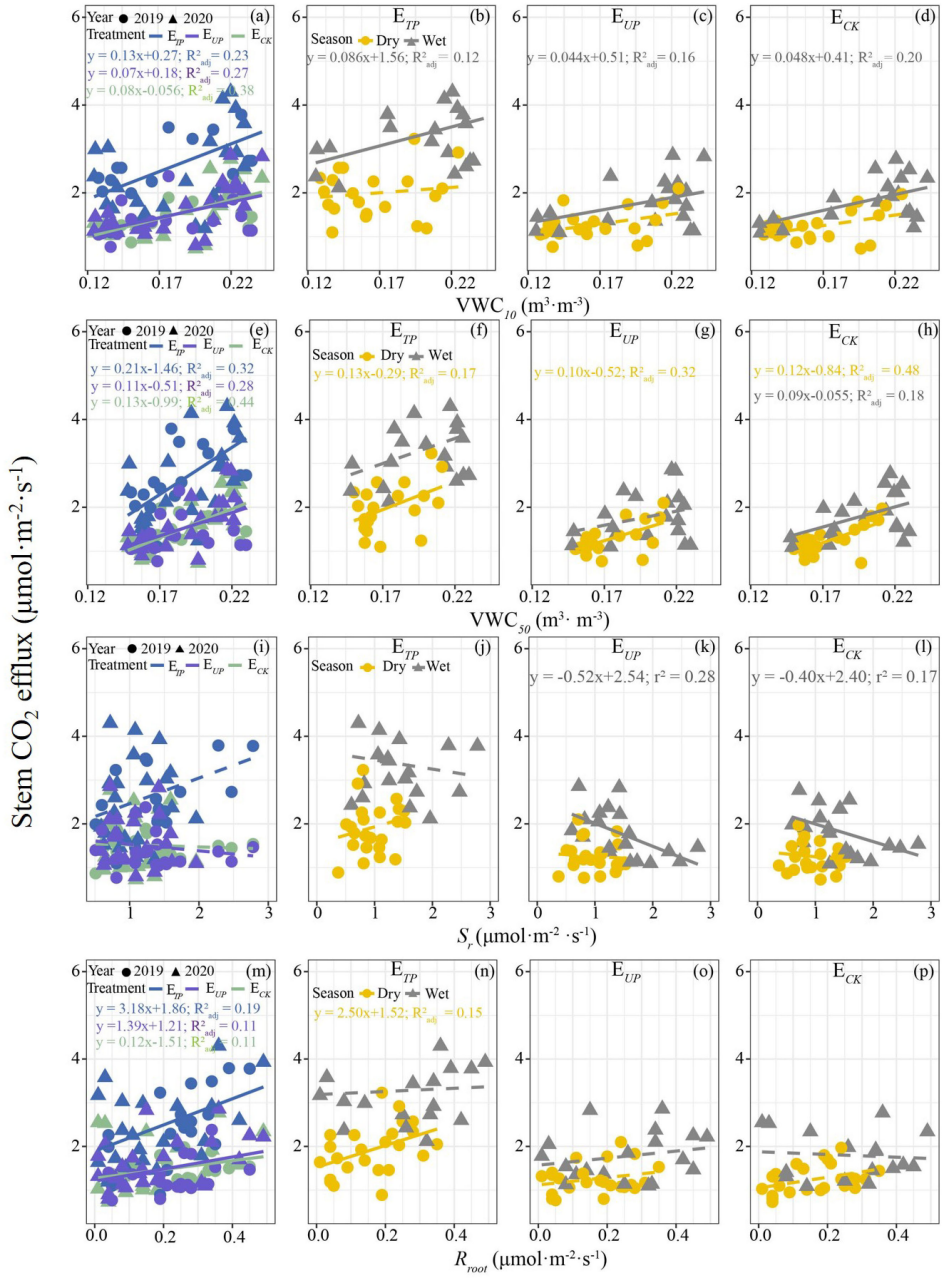
Relationships between *E*_c_ and soil moisture **(a-h)**, soil respiration **(i-l)**, and root respiration **(m-p)**. E*_TP_*, *E*_c_ in the tapped panel of tapping rubber trees; E*_UP_*, *E*_c_ in the untapped panel of tapping rubber trees; E*_CK_*, *E*_c_ in non-tapped rubber trees; VWC_10_, soil moisture at 10 cm soil depth; VWC_50_, soil moisture at 50 cm soil depth; S*_r_*, soil respiration; *R_root_*, root respiration. R^2^ and *p*-values for the relationships were calculated using a linear regression model. The solid line indicates a significant linear relationship between the two factors (*p* < 0.05), while the dotted line signifies the absence of such a relationship.

Overall, a clear linear relationship was observed between *E_c_* and soil temperature and moisture, LAI, *F_d_*, root respiration (*R_root_*) and air temperature (*p* < 0.05) but these showed seasonal divergence (**Figure 7**; **Supplementary Figures S1**, **S2**). In terms of environmental variables, while *E_c_* demonstrated a significant positive correlation with soil, air temperature and LAI on an annual scale, these relationships became non-significant in seasonal analyses (**Supplementary Figure S2**). Similarly, annual positive correlations with soil moisture (**Figures 7a, e**) shifted seasonally, only surface soil moisture (10 cm depth) correlated with *E_c_* in wet seasons (**Figures 7f–h**), whereas deep soil moisture (50 cm depth) showed this relationship in dry seasons (**Figures 7b–d**). Physiological factors exhibited analogous patterns. *E_c_* showed no annual correlation with soil respiration (**Figure 7i**) but a significant negative wet-season correlation (*p* < 0.05; **Figures 7k, l**). Conversely, *R_root_* and *F_d_* correlated positively with *E_c_* annually (**Figure 7m**; **Supplementary Figure S2I**) but exclusively during dry seasons (**Figure 7n**; **Supplementary Figure S2J**).

**Figure 8:**
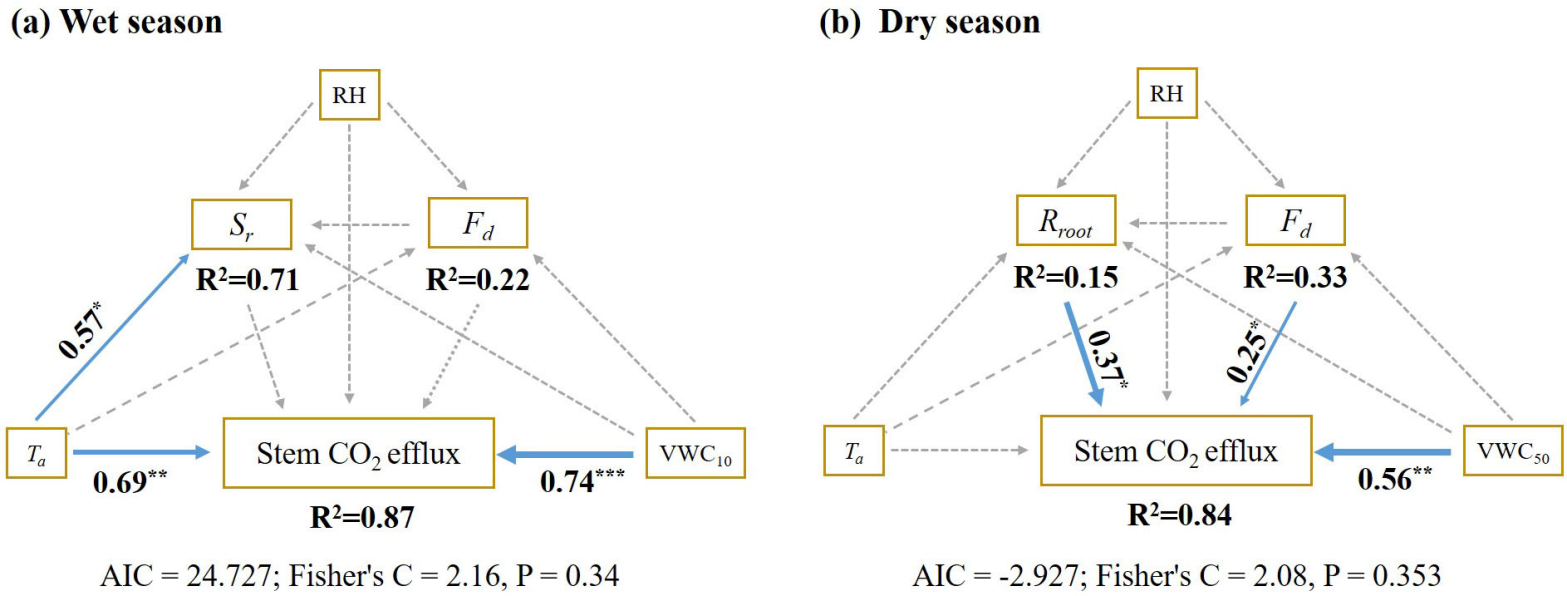
Piecewise structural equation models (SEM) for drivers of stem CO_2_ efflux variation during the wet season **(a)** and dry season **(b)**. RH, relative humidity; VWC*_10_*, soil moisture at 10 cm soil depth; VWC*_50_*, soil moisture at 50 cm soil depth; *T_a_*, air temperature; *F_d_*, sap flux density; *R_root_*, root respiratory; *S_r_*, soil respiration. Blue and Gray arrows represent a significant positive relationship and a non-significant relationship, respectively. Arrow width corresponds to the strength of the relationship, while the numbers adjacent to the arrows indicate standardized path coefficients. The goodness-of-fit statistics for the model are presented below. *p < 0.05; **p < 0.01; ***p < 0.001.

Structural equation models to uncover significant seasonal variations in the factors influencing stem CO_2_ efflux (*E_c_*). During the wet season, surface soil moisture at 5 cm soil depth and air temperature emerged as the primary factors directly affecting *E_c_* (**Figure 8a**). Conversely, in the dry season, the *E_c_* variations were predominantly influenced by belowground root respiration (*R_root_*), sap flow flux density (*F_d_*), and deep soil moisture content at 50 cm soil depth (**Figure 8b**). The model explain 87% and 84% of the variability in stem CO_2_ efflux during the wet season, respectively (**Figure 8**).

## 4 Discussion

### 4.1 Seasonal patterns in stem CO_2_ efflux in the rubber trees

Our results demonstrate distinct seasonal patterns in rubber *E_c_*, with significantly higher values during the wet seasons and reduced rates in dry seasons (**Figure 6**). This aligns with prior observations in rubber plantations (An et al., 2024), but contradicts patterns in other tree species (Zach et al., 2010; Ranniku et al., 2024). These discrepancies may reflect interspecific differences in growth phenology (Etzold et al., 2013; Macháčová et al., 2023). *E_c_* reached minima during the growth dormancy periods and increased with active growth. As we all know, leaf area index (LAI) dynamics reflecting canopy leaf flushing in early wet seasons and senescence in late dry seasons served as a reliable alternative indicator of growth phenology (Meir and Grace, 2002; Yang et al., 2016; Rogers et al., 2021). The significant positive correlation between LAI and *E_c_* throughout the study periods demonstrates that growth phenology regulates *E_c_* variation (**Supplementary Figure S2E**). Our data and previous studies have shown that rubber LAI and vegetation growth rate peaked during the wet season, while growth rate was slower during the dry season (**Figures 5a, b**; Liu et al., 2022). *E_c_* was generally highest when tree transpiration and stem growth rate were at their peak (Salomón et al., 2016; Chan et al., 2018), which is typically during a high rate of cell enlargement and growth phase, accompanied by high photosynthetic activity (Gordon and Larson, 1968; Hernández-Santana et al., 2021). As a result of this photosynthetic activity, carbohydrate concentration levels are likely to rise (Chan et al., 2018). Plant respiratory activity is believed to be influenced by substrate supply, respiratory product demand, and potential enzyme capacity (Zach et al., 2010). Consequently, rubber tree *E_c_* tends to be higher during the wet season when growth is rapid and lower during the dry season when growth is slower.

### 4.2 Response of rubber tree *E_c_* to tapping activities

Consistent with our first hypothesis, tapping activities increased stem CO_2_ efflux from rubber trees, and the magnitude of this increase showed clear seasonality. Notably, compared to the non-tapped trees, tapped trees exhibited a significant increase in *E_c_* on the tapped panel, without altering the seasonal trend and affecting *E_c_* on the untapped panel (**Figure 6**). The increase in CO_2_ efflux on the tapped panel of tapped rubber trees may be attributed to the following reasons: First, non-structural carbohydrates (NSC) are the precursor of rubber molecules and serve as energy for latex metabolism (Jacob et al., 1998), tapping increases the NSC accumulation on the tapping panel (unpublished data; Silpi et al., 2007; Chantuma et al., 2009). Increased NSC provides sufficient substrate supply for tree respiratory activities, potentially resulted in heightened *E_c_* (Maier et al., 2010; Rodríguez-Calcerrada et al., 2014). Second, a significant anatomical difference exist between renewed bark formed after tapping and virgin bark. Renewed bark had a higher proportion of soft to hard bark compared to virgin bark (Thomas et al., 1995). Functionally active tissue and conductive tissue were primarily concentrated in soft bark, while tissue in hard bark was less functional or shriveled (Bowman et al., 2005; Gopal and Thomas, 2014). In addition, renewed bark exhibits greater radial diffusion ability than virgin bark (Gopal and Thomas, 2014). This enhanced diffusion likely facilitates greater release of CO_2_ from the tapped panel of the tapping rubber trees. Consequently, alterations in the anatomical structure of the bark post-tapping contributed to increasing *E_c_* on the tapped panel. Third, tapping reduces sap flow density (Kunjet et al., 2013), increasing the likelihood of radial diffusion of CO_2_ dissolved in the transpiration stream. As a result, more CO_2_ from belowground respiration may more easily diffuse through the more conductive regenerated bark, thereby increasing stem CO_2_ efflux on the tapped panel of the tapping rubber trees.

Tapping induces a greater increase in stem CO_2_ efflux (*E_c_*) during the wet season compared to the dry season (**Figure 6**). This difference is likely attributed to tree phenology, the tapping intensity, and seasonal environmental factors (**Figure 7**; **Supplementary Figures S1, S2**). In the wet season, rubber trees experience active growth under favorable conditions (e.g., abundant water, optimal temperatures), associated with higher metabolic rates and increased respiration (**Figure 6**). Tapping wounds initiate energy-intensive processes such as cellular repair, tissue healing, and enhanced latex production (Biggs, 1992), demanding significant energy and elevating CO_2_ release through respiration. As trees are more metabolically active during the wet season, the energy required for these reparative processes is higher, leading to a greater rise in CO_2_ emissions. Conversely, during the dry season, water scarcity and lower temperatures slow tree growth and metabolic activity. In this context, the overall metabolic and repair processes decelerate, diminishing the impact of tapping on stem respiration and CO_2_ efflux.

### 4.3 Seasonal differences in the relationship between *E_c_* and belowground respiration

During the dry season, a significant linear relationship was observed between *E_c_* and root respiration, with belowground respiration identified as the primary driver of *E_c_* changes. In contrast, the wet season exhibited no similar correlation. These findings supported our second hypothesis. One potential explanation for this phenomenon is the variability in tree growth, transpiration, and other physiological activities resulting from the uneven rainfall distribution (Bužková et al., 2015; Salomón et al., 2024). Most stem CO_2_ originates from respiring cells in stems and roots (Teskey et al., 2008). Living cells reside in the inner bark, vascular cambium, and xylem, whereas the outer bark (rhytidome) lacks living cells (Rosell, 2019). Stem CO_2_ efflux derives from respiration in xylem, cambium, and inner bark, or is imported via the transpiration stream. CO_2_ diffuses radially through inner and outer bark via structures such as lenticels, cracks, and wounds (Teskey et al., 2008). Consequently, variations in the properties of the bark, cambium, lenticels, cracks, and wounds significantly impact CO_2_ diffusion. Bark and cambium activity exhibit significant seasonal changes (Rao and Rajput, 2001). During the wet season, vigorous growth metabolism drives active cambium cell division, generating new xylem and phloem cells (Teskey et al., 2008). These newly formed cells exhibit stronger metabolism and more efficient CO_2_ exchange than in the dry season. This seasonal metabolic pattern is indirectly supported by the stem CO_2_ seasonality we observed in our rubber trees.

Interestingly, no significant relationship was observed between root respiration and stem CO_2_ efflux during the wet season in our study, which may be due to the high transpiration (Dukat et al., 2024). In the wet season, frequent rainfall allows for the full replenishment of soil moisture, creating a hydrothermal synchronization conditions that foster rapid tree growth, metabolism and enhanced tree transpiration and respiration rates (**Figures 5**, **8b**). Previous studies demonstrate a significant negative correlation between sap flow rate and stem CO_2_ efflux under ample water availability (Bowman et al., 2005; Dukat et al., 2024). High transpiration rate facilitates transport of dissolved CO_2_ (from root respiration) to the canopy, reducing its radial diffusion opportunity. Consequently, root-derived CO_2_ contributes to stem CO_2_ efflux is minimal or negligible, leading to the observed lack of significant correlation (Bloemen et al., 2013a, b; Kumagai et al., 2015). Furthermore, during the wet season, root metabolism was more active. Hence, part of the CO_2_ produced by the root respiration may dissolve into soil water rather than xylem sap. Elevated temperatures also decrease the solubility of CO_2_ in water (according to Henry’s law), resulting in the release of CO_2_ from the soil to the atmosphere. This may partly explain both the lack of correlation between the *E_c_* and *R_root_* and the negative correlation with soil respiration during the wet season (**Figures 7k, l, n**). In contrast, during the dry season, inadequate rainfall does not sufficiently replenish soil moisture, with trees relying primarily on deeper soil water. Consequently, tree metabolism and transpiration rates slow significantly compared to those observed during the wet season (**Figure 5**). This prolongs retention of root-absorbed water within the stem (Bowman et al., 2005), providing greater opportunity for root-respired CO_2_ to diffuse radially into the atmosphere (Bloemen et al., 2013a). A recent study reported a positive correlation between sap flow flux density and the vertical transport of CO_2_ and pointed out that minimal sap flow density coincided with maximal contribution of axially transported CO_2_ to stem CO_2_ efflux (Dukat et al., 2024). Our findings partially elucidate the strong seasonal variability in how environmental factors explain tree *E_c_* (Etzold et al., 2013). Furthermore, our case emphasizes that the seasonal relationship between *E_c_* and belowground respiration results from complex interactions between environmental conditions and eco-physiological processes (Steppe et al., 2015; Yang et al., 2016; Tarvainen et al., 2018).

### 4.4 Implications

Our study conducted a paired experiment using tapped and non-tapped rubber trees as the subjects of research. This experimental design offers the advantage of comparing the impact of tapping activities on the *E_c_* of rubber trees. Additionally, the alterations in the structure of the regenerated bark of rubber trees post-tapping facilitate the diffusion of CO_2_ within the tree, enabling an investigation into whether stem CO_2_ efflux is influenced by belowground respiration. Our findings indicated that seasonal variations in the environment altered the role of belowground respiration in *E_c_* by changing physiological traits. These findings add to the growing body of evidence that transpiration rate and root respiration play an important role in regulating stem CO_2_ efflux (Bloemen et al., 2013a, b).

Furthermore, our findings suggested that *E_c_* reflected both stem and belowground (soil and root) respiration activity, with seasonal climate variations influencing the relative contributions of each to the *E_c_* (De Roo et al., 2019; Salomón et al., 2024). This study advances our mechanistic understanding of how climate-driven environmental variability modulates carbon allocation in rubber tree ecosystems, offering critical empirical evidence for refining carbon flux models under projected climate change scenarios.

## 5 Conclusion

A long-term investigation of stem CO_2_ efflux (*E_c_*) in both tapped and non-tapped rubber trees, in combination with an examination of environmental factors and physiological traits, enabled the study of seasonal *E_c_* patterns and underlying mechanisms. Our results suggested that the *E_c_* seasonal dynamics of rubber trees exhibited a single-peak pattern. This pattern may be attributed to the growth phenology of rubber trees, because in our study, although rubber tapping influenced *E_c_* magnitude, it did not significantly alter the overall seasonal trend. The relationships between stem CO_2_ efflux and belowground respiration varied significantly with season. These phenomena likely originated from seasonal fluctuations in tree metabolic activity and soil moisture driven by uneven annual rainfall distribution and environmental changes. During the wet season, rainfall was frequent, soil moisture was adequate, and tree transpiration was vigorous. High transpiration rate facilitated the rapid transport of root-derived CO_2_ to the canopy, thereby diminishing its contribution to *E_c_*. Conversely, in the dry season, reduced rainfall limited soil moisture recharge, leading to declines in tree metabolism and transpiration rate. This slower transpiration resulted in greater lateral diffusion of CO_2_ originating from root respiration through the stem to the atmosphere. Our results suggested a more complex and challenging linkage between belowground respiratory processes and *E_c_* than previously understood. Integrating seasonal *E_c_* dynamics with physiological traits will offer a more comprehensive approach to understanding how environmental and physiological factors interact to regulate stem CO_2_ efflux.

## Data Availability

The raw data supporting the conclusions of this article will be made available by the authors, without undue reservation.
