# Supplementary material for: Seasonal drought shapes the relationship between stem CO2 efflux and belowground respiration in an even-age rubber plantation on Hainan Island, China
Source: Front Plant Sci. 2025 Jul 16;16:1552859. doi: 10.3389/fpls.2025.1552859 (PMC12307319; doi:10.3389/fpls.2025.1552859)
Supplement: Supplementary file 1 [file DataSheet1.docx]

**Supplementary materials**

**Figure S1.** Relationships between *E*_c_ and soil temperature (a-h), air temperature (i-l), and relative humidity (m-p). E*_TP_*, *E*_c_ in the tapped panel of the tapped rubber trees; E*_CK_*, *E*_c_ in the untapped panel of the tapped rubber trees; E*_NP_*, *E*_c_ in the non-tapped rubber trees; *T_10_*, soil temperature at 10 cm soil depth; *T_50_*, soil temperature at 50 cm soil depth; *T_a_*, air temperature; RH, relative humidity. R^2^ and *p*-values for the relationships were calculated using a linear regression model. R² and p values for the relationships were derived from linear regression analysis. A solid line denotes a statistically significant linear relationship between factors (*p* < 0.05), whereas a dotted line indicates no significant relationship. The same below.

**Figure S2.** Relationships between *E*_c_ and vapor pressure deficit (a-d), leaf area index (e-h), and sap flux density (i-l). VPD, vapor pressure deficit; LAI, leaf area index; F*_d_*, sap flow flux density; RH, relative humidity.


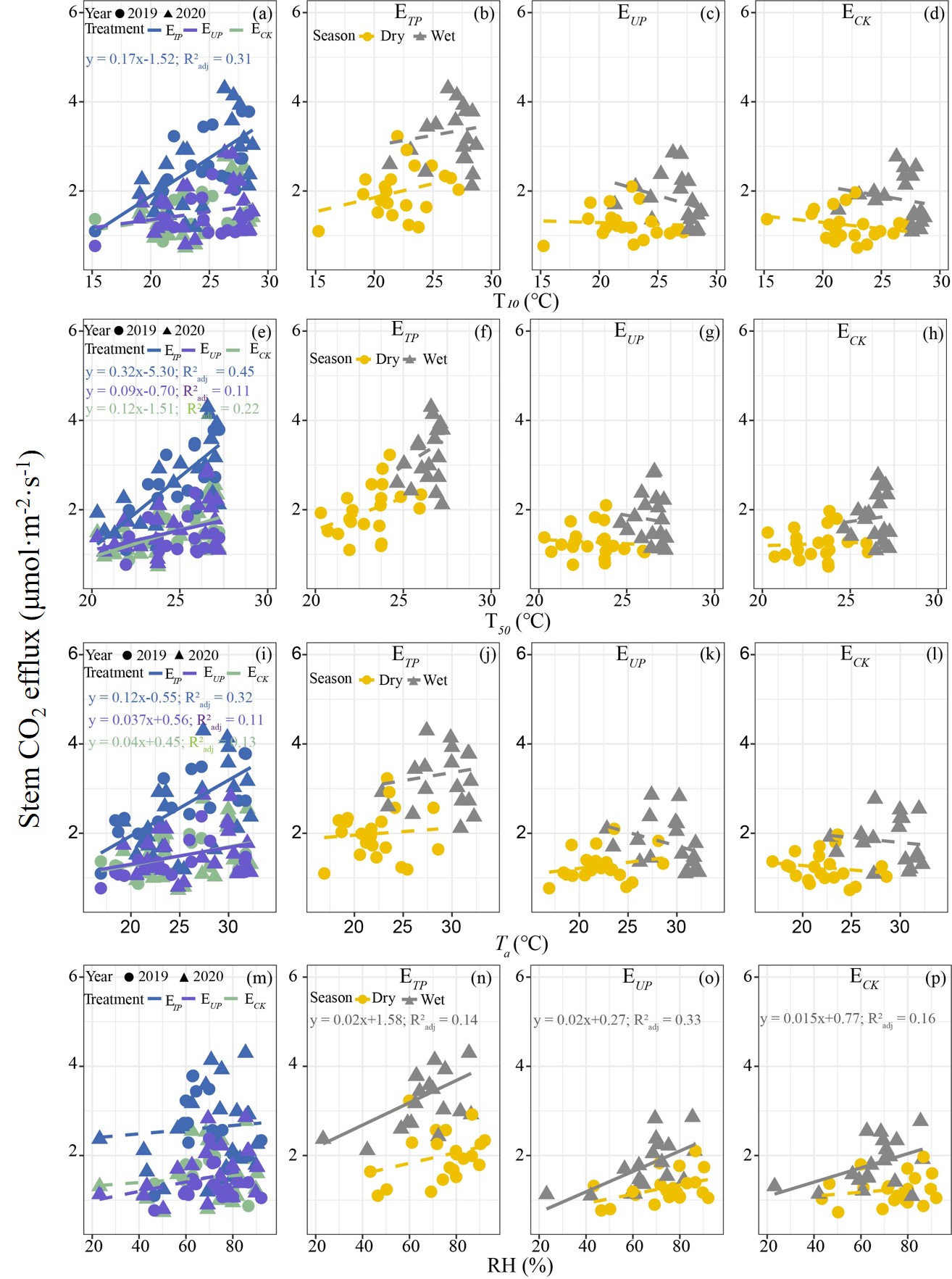


**Figure S1.** Relationships between *E*_c_ and soil temperature, *T_a_*, and RH.


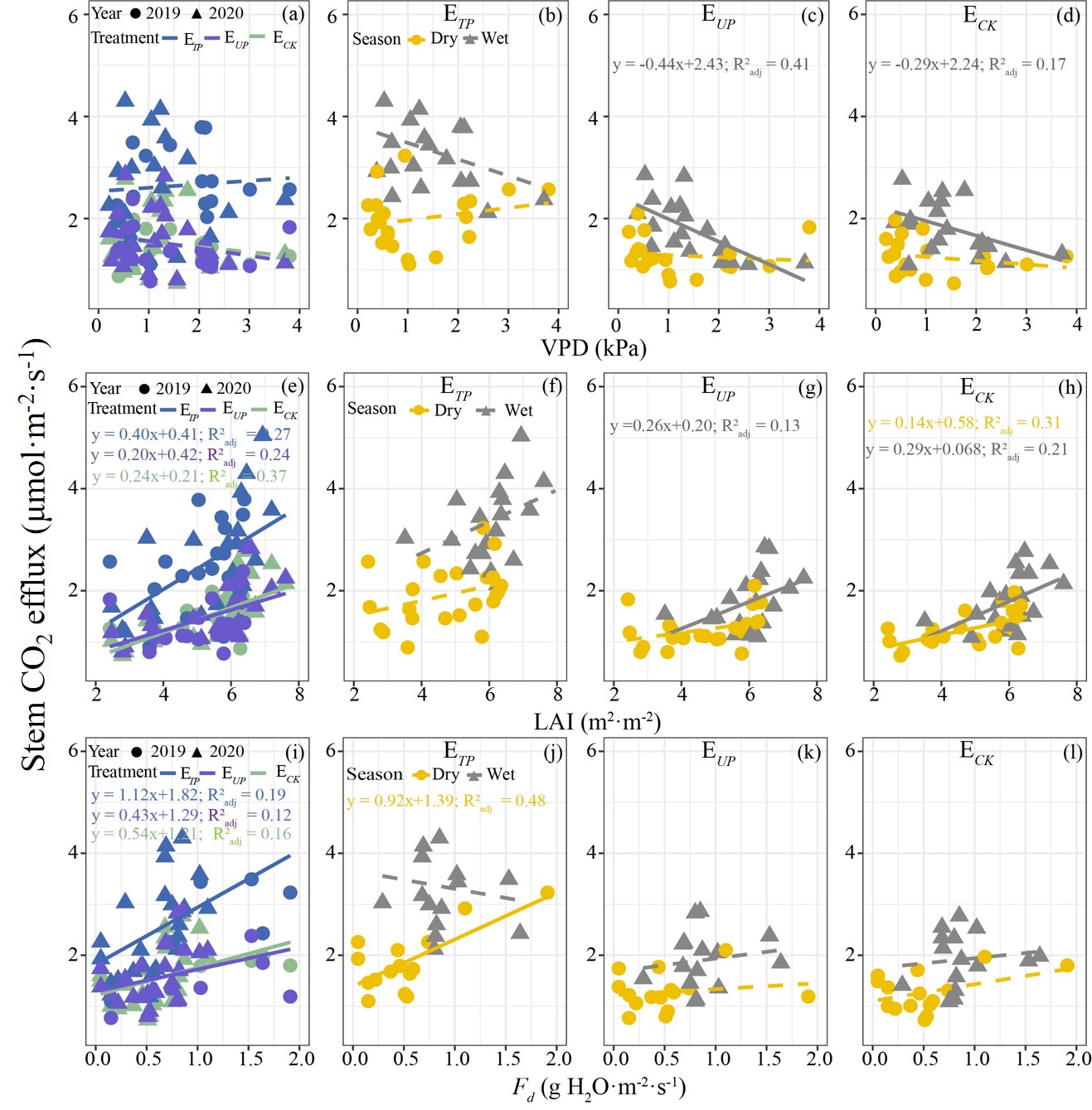


**Figure S2.** Relationships between *E*_c_ and VPD, LAI, and *F_d_*.
